# The Role of S-Glutathionylation in Health and Disease: A Bird’s Eye View

**DOI:** 10.3390/nu16162753

**Published:** 2024-08-18

**Authors:** Luca Federici, Michele Masulli, Vincenzo De Laurenzi, Nerino Allocati

**Affiliations:** 1 Department of Innovative Technologies in Medicine and Dentistry, University “G. d’ Annunzio”, 66100 Chieti, Italy; luca.federici@unich.it (L.F.); michele.masulli@unich.it (M.M.); vincenzo.delaurenzi@unich.it (V.D.L.); 2 CAST (Center for Advanced Studies and Technology), University “G. d’ Annunzio”, 66100 Chieti, Italy

**Keywords:** glutathione, GSH, S-glutathionylation, GS-ylation, oxidative stress, post-translational modification

## Abstract

Protein glutathionylation is a reversible post-translational modification that involves the attachment of glutathione to cysteine residues. It plays a role in the regulation of several cellular processes and protection against oxidative damage. Glutathionylation (GS-ylation) modulates protein function, inhibits or enhances enzymatic activity, maintains redox homeostasis, and shields several proteins from irreversible oxidative stress. Aberrant GS-ylation patterns are thus implicated in various diseases, particularly those associated with oxidative stress and inflammation, such as cardiovascular diseases, neurodegenerative disorders, cancer, and many others. Research in the recent years has highlighted the potential to manipulate protein GS-ylation for therapeutic purposes with strategies that imply both its enhancement and inhibition according to different cases. Moreover, it has become increasingly evident that monitoring the GS-ylation status of selected proteins offers diagnostic potential in different diseases. In this review, we try to summarize recent research in the field with a focus on our current understanding of the molecular mechanisms related to aberrant protein GS-ylation.

## 1. Introduction

Post-translational modifications are reversible or irreversible events that change the properties of a protein after its biosynthesis [1]. They are involved in several biological processes such as DNA repair, signal transduction, apoptosis, and cell cycle control [1]. The interruption or misregulation of such modifications can lead to the dysfunction of biological processes, and it is thus associated with several diseases. In particular, post-translational modifications of thiol groups, such as S-glutathionylation (GS-ylation), S-nitrosylation and S-sulfuration, play an important role in cellular responses to oxidative stress.

GS-ylation is an important and ubiquitous post-translational reversible modification resulting in mixed disulfides between glutathione and the cysteine residues. It is recognized as one of the most important post-translational mechanisms that—under physiological conditions—regulates various cellular processes [2,3,4,5,6]. In pathological conditions, abnormal GS-ylation is associated with cardiovascular, pulmonary, and neurodegenerative diseases as well as cancer and diabetes [5]. The amino acid cysteine contains a sulfhydryl group (SH, referred to also as thiol) in its side chain and the thiolate group—the anionic deprotonated form of the thiol—is one of the most reactive and ubiquitous intracellular nucleophiles in biological systems. Not surprisingly, despite its low abundance as compared to other amino acids, 90% of cysteine residues are highly conserved within protein sequences among species [7,8].

**Figure 1:**
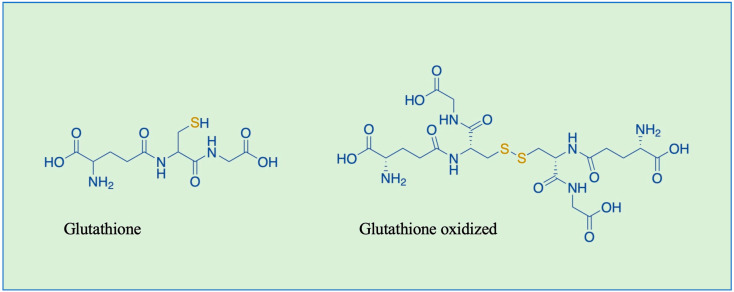
Chemical structures of glutathione (GSH) and glutathione oxidized (GSSG).

Glutathione (L-γ-l-glutamyl-L-cysteinyl-glycine) is the most abundant eukaryotic and prokaryotic low molecular weight thiol, with cellular concentrations in the millimolar range, and it is a main regulator of redox status and redox signal transduction within cells [9,10,11]. Glutathione is present in all organs—liver, in particular—and it exists inside the cells in both reduced (GSH) and oxidized (GSSG) forms (Figure 1). Under physiological conditions, the intracellular GSH/GSSG ratio is higher than 100:1 to maintain intracellular redox homeostasis, but it decreases by 10 folds or less under oxidizing conditions. Thus alterations in the cellular GSH/GSSG ratio are often monitored as a biomarker of oxidative stress [12,13]. The GSH sulfhydryl group is able to react with and neutralize both exogenous and endogenous harmful molecules, such as reactive oxygen species (ROS), reactive nitrogen species (RNS), and xenobiotics [9,14]. ROS are oxygen-derived molecules including superoxide anions, hydrogen peroxide, and hydroxyl radicals. The generation of ROS is induced by extracellular and intracellular stimuli as well as during mitochondrial oxidative metabolism, the primary source of endogenous ROS [15,16,17]. RNS are nitric oxide-derivative compounds that play a crucial role in the physiologic regulation of many living cells [18]. High levels of ROS and/or RNS are implicated in cell injury and death by inducing oxidative or nitrosative stress. In this review, we try to provide a comprehensive and updated overview of the state of the art in the field of GS-ylation research, with a particular focus on recent studies highlighting the involvement of protein GS-ylation in disease.

## 2. Literature Search

Although this work is a “narrative review” that aims to be mainly descriptive and does not involve a systematic search of the literature, the cited articles were retrieved following the guidelines for systematic reviews. We searched for articles published up to 9 April 2024 in the PubMed and Web of Science databases, the Google scholar search engine, and the social network ResearchGate. There were no restrictions on the language or region. We used several keywords for retrieving cited articles, such as “S-glutathionylation” and “physiological roles”, or “S-glutathionylation” and “(several) disease”. We have retrieved the complete list of abstracts, and the papers that followed the defined criteria for this study were reviewed in full. Additional articles were manually searched by checking the references lists of already included papers. Two authors (N.A. and L.F.) independently examined all the search results.

## 3. S-Glutathionylation Physiological Role

**Figure 2:**
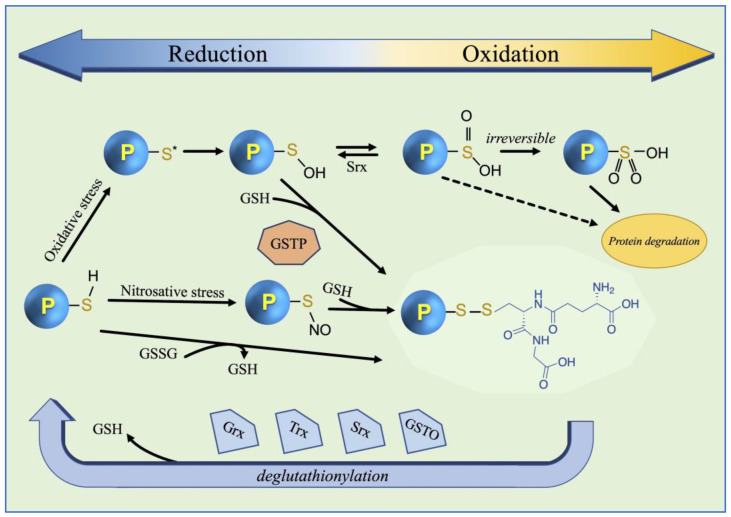
Forward and reverse reactions of the protein GS-ylation cycle. Cysteines can be oxidized into sulfenic acid (-SOH), sulfinic acid (-SO_2_H), and sulfonic acid (-SO_3_H). The hyperoxidized form of sulfinic acid can be reduced by sulfiredoxin (Srx) [31]. RNS can react with thiols to form S-nitrosylation (-SNO) and GSSG can form GS-ylation (-SSG) with reactive thiols. S*: thiyl radical.

Under physiological conditions, GS-ylation is involved in cellular signaling events and in the redox regulation of protein functions [2,19]. In a recent study, it was hypothesized that GS-ylation also plays a role in maintaining cellular circadian biorhythms [20]. In the cytosolic reducing environment, the sulfhydryl groups of protein cysteines retain a pKa > 8 and are chiefly in a protonated form and consequently not sensitive to oxidation. In addition to the pKa value of the target cysteine, GS-ylation is determined by other factors such as the accessibility of the cysteine sulfhydryl group to GSH and the stability of the resulting disulfide, which is in turn a function of the chemical environment [21]. For instance, a rapid reduction of the disulfide can be caused by a second cysteine nearby, as happens in the active sites of glutaredoxin (Grx) or thioredoxin (Trx). The GS-ylation of proteins serves as a protective mechanism against irreversible oxidative or nitrosative damage as well as a means of GSH storage [16,22]. It protects protein cysteines from being overoxidized by several mechanisms such as the reduction of sulfenic acid, thiol–disulfide exchange status (GSH/GSSG) or through the formation of S-nitroso thiols (Figure 2). The process of GS-ylation can be either spontaneous—although uncommon—or proceed by the catalytic activity of enzymes, mainly glutathione transferases of the Pi class (GSTP) [3,22,23,24,25,26]. The role of human glyoxalase II in the mechanism of GS-ylation was also observed by using an in silico approach [27]. The addition of GSH to target proteins leads to an increase in the molecular mass and an additional negative charge (due to the glutamic acid residue), and a consequent change in protein conformation and stability, altering its function in the physiological process. Effects of the GS-ylation have been observed on several enzymes altering their functionality. The addition of GSH may change the enzymatic catalytic properties (inhibition or increase in the activity), affect their structural stability, and alter ligand binding [28]. The rate of GS-ylation is dramatically enhanced by GSTP which is present primarily in the cytosol but also in other subcellular compartments [3]. The reverse reaction, de-GS-ylation, is mainly mediated by three members of the oxidoreductase family such as Grx, Trx, and sulfiredoxin (Srx) (Figure 2) [22,23,29,30,31]. Similar functions were also attributed to a GST of the Omega class (GSTO) [32]. Occasionally Grx can also catalyze protein GS-ylation [33,34]. GS-ylation also has a key role in cellular detoxification through GSTs. GSTs metabolize a wide variety of xenobiotic and endobiotic electrophilic compounds and their metabolites, including carcinogens, drugs, environmental pollutants, and pesticides, via GSH conjugation. This reaction is the first step in mercapturic acid formation, a pathway through which harmful molecules are inactivated and eliminated from an organism [35].

## 4. S-Glutathionylation in Disease

Deregulated protein GS-ylation has been associated with neurodegeneration, diabetes, cardiovascular diseases, and cancer. Although reversible, GS-ylation may become pathologic during prolonged oxidative stress. Indeed, under pathological conditions, an increase in protein GS-ylation is often associated with an abnormal production of oxidative stress leading to cell damage, due to the oxidation of proteins, DNA, and lipids. Under oxidative stress conditions, ROS concentrations increase and, because of their high reactivity, they take part in several chemical reactions [36].

### 4.1. Cardiovascular Diseases

GS-ylation is a critical signaling mechanism in cardiovascular physiology because it regulates several processes involved in homeostasis [36]. Therefore, perturbations in protein GS-ylation status may contribute to the etiology of many cardiovascular diseases, including myocardial infarction, cardiac hypertrophy, and atherosclerosis, as detailed below.

#### 4.1.1. GS-Ylated Na^+^-K^+^ Pump

*Caveolae* are invaginations of the plasma membrane and they are found in various vertebrate cell types and also in cardiovascular system tissues such as blood vessel endothelial cells, smooth muscle cells, and cardiomyocytes [37]. Caveolins are a family of integral membrane proteins that constitute the main structural components of caveolae. Caveolae participate in several physiological processes like the uptake of several molecules and are involved in pathophysiological changes in the cardiovascular system [38,39]. GS-ylation is critical in regulating cardiovascular functions like cardiomyocyte contraction, as well as in mediating signaling events affecting protein synthesis within the cardiovascular system [36,39]. The GS-ylation of the ion pump Na^+^-K^+^ ATPase may act as a protective response to oxidative stress. Under conditions of increased ROS, the modification prevents the irreversible oxidation of critical cysteine residues, thereby protecting the pump from permanent damage [40]. In the *caveolae*, the GS-ylation of the ion pump Na^+^-K^+^ ATPase b_1_ subunit has been identified as a key mechanism for pump inhibition, with significant implications for diseases distinguished by high oxidative stress, specifically in the cardiovascular system [39]. 

#### 4.1.2. GS-Ylation of cMyBP-C

Cardiac myosin binding protein-C (cMyBP-C) is a sarcomeric protein located on the thick filament, mainly involved in the regulation of contraction and relaxation controlled via cMyBP-C phosphorylation, whose ablation has been frequently associated with heart failure. It has been observed that reduced cMyBP-C phosphorylation corresponded to increased GS-ylation in ventricular tissue from patients with dilated or ischemic cardiomyopathy compared to controls [41]. The reduction of phosphorylation of cMyBP-C led to increased injury and subsequently to dysfunction. Using the redox proteomics technique, a cysteine cluster in the proximity of the regulatory phosphorylation sites within the C1-M-C2 domain was identified, showing enhanced GS-ylation in patients [41].

It was also shown that elevated myocardial S-glutathionylated cMyBP-C—due to an increase in oxidative stress—is correlated with diastolic dysfunction, a pathological condition that can lead to heart failure and the consequent increase in mortality (Table 1) [42,43]. Table 1 summarizes some examples of disease-relevant proteins that are GS-ylated. S-glutathionylated cMyBP-C has been studied as a biomarker for cardiac diastolic dysfunction [42]. The results obtained support the hypothesis that circulating S-glutathionylated cMyBP-C can serve as a diagnostic biomarker. These are important results considering that the standard diagnosis is the echocardiographic evaluation which is generally used in symptomatic patients. Conversely, the S-glutathionylated cMyBP-C test could be used in asymptomatic people at risk of heart failure.

#### 4.1.3. Hypertrophic Cardiomyopathy

Hypertrophic cardiomyopathy (HCM) a congenital genetic heart disease and is one of the most common causes of sudden cardiac death in young people. Among the genes with mutations associated with HCM is *cMyBP-C* (about 40% of frequency) [44]. Diastolic dysfunction is a common feature of both HCM and heart failure with preserved ejection fraction (HFpEF). It has been hypothesized that oxidative stress is a key mechanism for the development of abnormal diastolic function. Particularly, the GS-ylation of cMyBP-C has been identified as an important player in the development of diastolic dysfunction. Furthermore, a notable increase in GS-ylation in models of HCM and HFpEF has been observed [45]. S1P (sphingosine-1-phosphate) is a signaling sphingolipid that has a significant role in cardiovascular and muscle biology. FTY720 (fingolimod) is a small-molecule S1P analog, modulator of the S1P receptor, currently in use for the treatment of multiple sclerosis. It has been observed that FTY720 administration in a mouse model of HCM, resulted in a partial reversal of diastolic dysfunction and a reduction in left atrial enlargement, and these changes were associated with decreased GS-ylation of cMyBP-C [45].

#### 4.1.4. GS-Ylation of Rac1

Metabolic disorders, such as diabetes mellitus and hyperlipidemia, facilitate cardiovascular diseases. Oxidative stress is involved in the rise of vascular permeability associated with metabolic disorders. In the endothelial cells, the correct functionality of the cell barrier is coordinately governed by Rho GTPases, including Rac1. Rho GTPases are molecular switches that control a broad range of signal transduction pathways [46]. They have a key role in regulating the actin cytoskeleton architecture and they are involved in the regulation of many other signal transduction pathways [46,47]. Metabolic stress-induced GS-ylation in human aortic endothelial cells was correlated with high cell permeability [48]. Furthermore, in endothelial cells isolated from patients with type-2 diabetes mellitus, Rac1 GS-ylation levels were increased and correlated with cell hyperpermeability. GS-ylation and the following inactivation of Rac1 in endothelial cells represent a novel redox mechanism of vascular barrier dysfunction associated with metabolic disorders [48].

#### 4.1.5. Cerebrovascular Diseases

Cerebrovascular diseases represent a subset of cardiovascular diseases, consisting of different and multifactorial disorders of the blood vessels supplying the brain such as stroke, stenosis, and aneurysms. Nitric oxide synthase (NOS), which converts L-arginine to L-citrulline and NO, is an important mediator of cellular signaling and promotes vascular smooth muscle relaxation. Endothelial nitric oxide synthase (eNOS) is located in the *caveolae* and represents the prominent enzymatic source of NO in the vascular wall. Under oxidative stress, eNOS activity was reported to decrease through GS-ylation [49]. The GS-ylation of eNOS causes functional uncoupling, the reduction of NO synthesis, and increased superoxide anion generation. In human eNOS, two highly conserved cysteine residues were identified as sites of GS-ylation and found to be critical for redox regulation of the enzyme function [49]. Cerebral ischemia is a condition that occurs when there is an insufficient amount of blood flow to the brain. Following brain ischemic injury, a combined administration of L-citrulline and GSH was shown to inhibit eNOS GS-ylation improving the function of the enzyme in brain [50].

### 4.2. Vascular Diseases

Endothelial cell-to-cell junctions are adhesive intercellular contacts which maintain and regulate normal microvascular function. Furthermore, they act as signaling units that modulate several functions including protection from apoptosis and inhibition of cell growth. Modifications in the adherens junction impact endothelial cell motility, vascular morphogenesis, and permeability. Vascular endothelial (VE)-cadherin—a member of the cadherin family—is the main endothelial-specific adhesion protein at cell junctions [51]. Cadherins, [Ca (calcium) + adherin (to adhere)], are a family of membrane glycoproteins essential in maintaining cell-to-cell contact and regulating cytoskeletal complexes. Cadherins are also involved in several pathological functions ranging from cancer progression and metastasis to being entry receptors for pathogens [51,52]. The phosphorylation of VE-cadherin/catenin complex proteins and subsequent internalization of VE-cadherin may disorganize the cell junctions, increasing the permeability of the endothelium and allowing the entry of toxic substances as well as pathogens [53]. It has been demonstrated that GSTP inhibits the phosphorylation of VE-cadherin through the GS-ylation of Src—the upstream tyrosine kinase of VE-cadherin—maintaining the endothelial barrier function [53]. VE-cadherin is a substrate for Src tyrosine kinase. Src family tyrosine kinases are non-receptor tyrosine kinases that are recognized as proto-oncogenic products. They are regulatory proteins that have a significant role in the disorganization of cadherin-dependent cell-to-cell junctions [54]. Thus, GSTP may act as negative regulator, ensuring appropriate vascular endothelial permeability and maintaining the functionality of the vascular endothelial barrier to prevent inflammation-related endothelial barrier disruption [53].

#### GS-Ylation of SirT1

Recently, it has been observed that the GS-ylation of sirtuin-1 (SirT1) is involved in the pathogenesis of thoracic aortic aneurysms associated with Marfan syndrome [55]. SirT1 is a member of the NAD (+)-dependent histone deacetylase family of sirtuins that function in the cellular response to inflammatory, metabolic, and oxidative stressors [56]. Previously, it was shown that vascular smooth muscle SirT1 is essential for the prevention of aortic dissections [57]. Thoracic aortic aneurysms—abnormal aortic dilatations—and aortic dissections or ruptures are the major cardiovascular complication of Marfan syndrome, a rare hereditary connective tissue disorder that causes changes in the bones, eyes, lungs, central nervous system, heart, and blood vessels. It has been shown that oxidative stress is increased in aneurysmal aortas in patients with Marfan syndrome. Furthermore, reversible oxidative post-translational modifications of protein cysteines, particularly GS-ylation, were dramatically increased in the aortas of a Marfan syndrome mouse model. It has also been observed that the GS-ylation of SirT1 inhibits its activity contributing to the onset of thoracic aortic aneurysm in Marfan syndrome. Interestingly, this led to the suggestion that, since no targeted therapy has been developed to date for Marfan syndrome, the prevention or reversal of the SirT1 GS-ylated state may represent a new therapeutic strategy to prevent the dissection/rupture of the TAA in these subjects [55].

### 4.3. Neurodegenerative Diseases

Neurodegenerative disorders are characterized by the progressive loss of selected populations of neurons associated with the formation of aberrant proteins with unstable structures that accumulate primarily in the brain and the spinal cord. The imbalance of redox homeostasis—resulting in increased oxidative stress—is considered a key risk factor that makes the brain vulnerable to aging and progressive neurodegeneration. Furthermore, the human brain is extremely susceptible to oxidative stress due to its high consumption of the total basal oxygen budget (about 20%) to support ATP-intensive neuronal activity, its shortage of antioxidants compared to other organs, as well as its lack of cellular regenerative capacity [58]. The most common neurodegenerative diseases are Alzheimer’s disease, Parkinson’s disease, Huntington’s disease (HD), amyotrophic lateral sclerosis (ALS), and Friedreich’s ataxia (FRDA).

#### 4.3.1. Alzheimer’s Disease

Alzheimer’s disease (AD) is a chronic neurodegenerative disease characterized by the pathological accumulation of β-amyloid peptides and neurofibrillary tangles—aggregates of hyperphosphorylated tau protein—in the brain, that usually starts slowly and gets worse over time up to dementia [59]. Although the exact cause of AD is not yet fully understood, it is becoming evident that oxidative stress plays a key role in the pathogenesis of the disease leading to neuronal cell death. The early identification of AD with conventional biomarkers—the ATN framework: A is amyloid, T is phosphorylated tau, and N is neurodegeneration—is problematic because they may overlap with the natural aging process. Indeed, β-amyloid peptides and tau proteins are frequently present at high physiological levels in elderly people. Many other elements are involved in AD. It has been observed that there is a positive correlation between two lipid peroxidation markers, hydroxynonenal and malondialdehyde, and β-amyloid [60]. At low concentrations, the β-amyloid peptide functions as a strong metal chelating antioxidant. On the contrary, when the β-amyloid peptide concentration increases, the protein evolves into a pro-oxidant causing an increase in oxidative stress and lipid peroxidation. The measurement of lipid peroxidation markers and redox status over time would be helpful in evaluating AD progression [60]. Furthermore, it has been observed that the altered redox status and subsequent increase in lipid peroxidation could contribute to the GS-ylation of proteins.

One of the earliest signs of AD is a reduction of glucose metabolism in the brain [61]. Neurons require large amounts of energy and metabolize glucose—their main source of energy—through glycolysis for ATP synthesis [62]. Glyceraldehyde 3-phosphate dehydrogenase (GAPDH) is a tetrameric cytosolic enzyme that catalyzes the oxidative phosphorylation of glyceraldehyde-3-phosphate to 1,3-diphosphoglycerate in the payoff phase of glycolysis. The GS-ylation of the active site of the sulfhydryl groups of GAPDH inhibits the enzymatic activity, resulting in a slowdown of glycolysis. It has been observed that under oxidative stress conditions, depletion of intracellular ATP—following inhibition of glycolysis—was almost entirely due to GAPDH inactivation [63]. New data provide a molecular rationale of how oxidative stress increases S-glutathionylated GAPDH in neurodegenerative diseases and this could lead to the identification of new therapeutic targets [63].

Using the redox proteomics approach, four proteins were identified, i.e., deoxyhemoglobin, α-crystallin B, GAPDH, and α-enolase, as significantly S-glutathionylated in AD brain samples compared with controls [64]. GAPDH was also shown to have reduced activity in the AD specimens compared with controls. The loss of protein activity consequent from oxidative modification could be a realistic mechanism for neurodegeneration found in the AD brain [64].

In addition to its key role in glycolysis, GAPDH is also involved in many other cellular functions unrelated to glucose metabolism and regulated through oligomerization, post-translational modifications, and subcellular localization. Several studies have shown that GAPDH is located in amyloid plaques and binds the amyloid precursor protein and the β-amyloid peptide, indicating that this protein might play a role in the progression of AD [65]. Furthermore, it has been observed that the oxidation of SH-groups in the active site of GAPDH produces non-native protein forms (both dimers and monomers) that translocate from the cytoplasm to the nucleus, followed by the development of apoptosis [66]. It has been supposed that the GS-ylation of GAPDH could prevent the dissociation of the enzyme and the accumulation of non-native GAPDH in the nucleus.

Significantly higher levels of S-glutathionylated GAPDH were found in the blood of AD patients compared to healthy controls [67]. It was proposed that the increasing accumulation of the β-amyloid precursor protein and β-amyloid peptide caused GAPDH oxidation. With neuronal cell death due to AD, GS-ylated GAPDH is released into the blood plasma [67]. These results suggested that the degree of neuronal apoptosis during AD progression might be evaluated based on the levels of GS-ylated GAPDH in the blood, which could be investigated as a new and more sensitive biomarker for this pathology [67].

The tumor-suppressor protein p53 is a DNA binding transcription factor that activates genes responsible for cell cycle arrest or apoptosis, in response to the exposure to stressors. Usually, the p53 protein is expressed at very low levels in normal cells. A p53 response can be activated by several types of stress, including DNA damage, oxygen deprivation, and variations in nutrient levels, supporting cell survival and defense from tumorigenesis [68]. After being imported into the nucleus and tetramerized, p53 proteins acquire the ability to bind with their target genes to induce tumor-suppressive responses [69]. p53 is also subject to numerous post-translational modifications, including GS-ylation [24]. It was reported that the GS-ylation of p53 may prevent the formation of the tetramer, and may be involved in oxidative stress conditions and neurodegeneration observed in AD pathogenesis [70]. The authors demonstrated elevated levels of GS-ylated p53 in the monomeric and dimeric forms in AD samples compared to controls. The GS-ylation of these two p53 forms prevents the formation of the tetramer and consequentially its functionality [70].

#### 4.3.2. Parkinson’s Disease

**Parkinson’s disease** (PD) is the second most common neurodegenerative disease after Alzheimer’s disease. PD is a long-term neurodegenerative disorder that affects the motor system, characterized primarily by the death of dopaminergic neurons in the *substantia nigra* and the presence of intracellular inclusions containing aggregates of misfolded α-synuclein (Lewy bodies) in the surviving neurons [71]. PD is also affected by changes in oxidative balance. A low concentration of GSH is considered to be one of the initial changes contributing to the onset of PD in the dopaminergic neurons of the *substantia nigra*, and is due to the formation of GSSG and glutathionyl conjugates, without diminution of GSH synthesis [72].

GSTP is a member of the superfamily of GSTs which is composed of multifunctional proteins, widely distributed in nature and primarily involved in phase II detoxification via GSH conjugation [14,73]. In addition to their key role in detoxification, GSTs are involved in several other catalytic and non-catalytic functions, including cell protection against oxidative stress [14,73]. Nuclear factor erythroid 2-related factor 2 (Nrf2) is a crucial transcription factor regulating the cellular response against oxidative and electrophilic stresses. Kelch-like ECH-associated protein 1 (KEAP1) is the most prominent regulator of Nrf2 activity. Under physiological conditions, KEAP1 sequesters Nrf2 and mediates its degradation. Under stress conditions, the reactive cysteine residues on the KEAP1 could be oxidized, leading it to lose its function, facilitating the escape of Nrf2 from degradation and nuclear accumulation [74]. In the nucleus, Nrf2 up-regulates the expression of several genes implicated in antioxidant defense, including GSTs. It has been observed that under oxidative stress, GSTP enhances the rate of GS-ylation of Keap1 leading to Nrf2 activation and subsequently increases the expression of the same enzyme. This positive feedback regulatory loop mechanism causes antioxidant protection in the brain [75].

**Figure 3:**
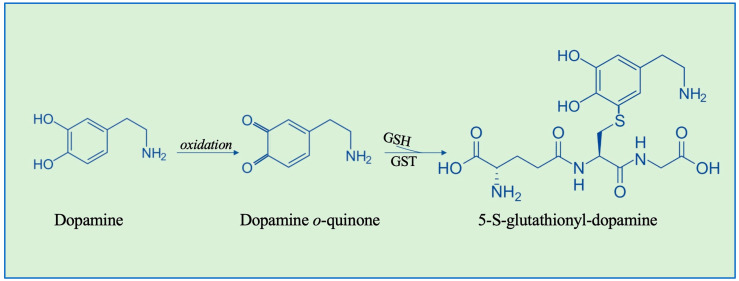
GS-ylation of dopamine catalyzed by mu class GST.

Dopamine oxidation, through the endogenous production of various neurotoxic metabolites—such as dopamine quinones and thio-catecholamines—triggers a cascade of molecular events leading to cell death, with a high specificity for dopaminergic neurons in the *substantia nigra*. The typical thio-catecholamines are 5-S-glutathionyl-dopamine and 5-S-cysteinyl-dopamine conjugates. 5-S-glutathionyl-dopamine was found to be resistant to oxidation by biological oxidizing agents; it has been therefore supposed that conjugation can serve as a neuroprotective antioxidant function [76]. This reaction may be catalyzed by GSTs. Indeed, it has been shown that a human GST of the mu class is able to conjugate dopamine with GSH (Figure 3) [76]. However, 5-S-glutathionyl-dopamine is (rapidly) enzymatically converted into the highly neurotoxic 5-S-cysteinyl-dopamine molecule and its benzothiazines that may cause severe damage to dopaminergic neurons [77]. 5-S-cysteinyl-dopamine is a key metabolite of high relevance for the early detection of PD and, recently, a new SERS-based method to detect 5-S-cysteinyl-dopamine as a biomarker for the diagnosis of PD has been developed [78]. 

#### 4.3.3. Huntington’s Disease

Huntington’s disease (HD) is a highly penetrant neurodegenerative disease caused by an autosomal dominantly inherited CAG trinucleotide repeat expansion in the huntingtin gene on chromosome 4. It is characterized by motor, cognitive, and behavioral abnormalities [79]. At the cellular level, the mutant huntingtin protein results in neuronal dysfunction and death through several mechanisms. It has been reported that HD patients have an increased level of oxidative stress markers—i.e., reduction of GSH concentration or aberrant calcium ions homeostasis—accompanied by a reduction in antioxidant systems compared to controls [80]. Transient receptor potential (TRP) channels are widely expressed multifunctional signaling molecules and function in several physiological processes. TRPC5, a member of the superfamily of TRPs is one of the calcium channels and it is primarily expressed in the brain. The effect of glutathionylated TRPC5 on striatal neurons in HD has been investigated [81]. Under oxidative stress, the GS-ylation of TRPC5 induces a significant enhancement of calcium ions in the cytosol, causing the activation of calmodulin-dependent protein kinase and the calpain–caspase pathway, thus leading neuronal cells to death [81].

#### 4.3.4. Amyotrophic Lateral Sclerosis

Amyotrophic lateral sclerosis (ALS) is a rare neurological disease that affects motor neurons. It was found that the increase in the GS-ylation of stromal interaction molecule 1 (STIM1), due to an oxidative environment, causes the dysregulation of Ca^2+^ entry—through an anomalous increase in store-operated Ca^2+^ entry (SOCE)—and endoplasmic reticulum (ER) calcium overload in astrocytes [82]. This leads to the subsequent excessive release of ER calcium into the cytoplasm. The ER membrane protein STIM1 sensor supervises ER-luminal Ca^2+^ levels to maintain cellular Ca^2+^ balance and to support Ca^2+^ signaling [83]. SOCE is a main Ca^2+^ influx mechanism whose activation is triggered by STIM1. It is known that STIM1 deficiency impairs calcium influx. Furthermore, it has been reported that altered STIM1 levels could support several pathologies, including immunodeficiency, cancer, and neurodegenerative diseases [84,85,86]. In regulated exocytosis, under the physiological state, the Ca^2+^ sensor is required to trigger the final stage of the fusion reaction [87]. In astrocytes, intracellular calcium levels are critical to the release of important active factors that modulate synaptic functions. It has been observed that altered calcium signaling causes abnormal exocytosis in an ALS astrocyte model contributing to the pathogenesis of ALS [82].

#### 4.3.5. Multiple Sclerosis

Multiple sclerosis (MS) is a chronic neurodegenerative disease of the central nervous system characterized by a combination of inflammation, demyelination, and axonal damage, with an unpredictable course of disease progression, which is associated with high oxidative stress. Using a modified proteomics approach, comparing cerebrospinal fluid samples in MS patients versus controls, eight GS-ylated proteins were identified and characterized in subjects with MS. Three of them were GS-ylated at the functional cysteine residues, resulting in their functional perturbation [88].

#### 4.3.6. Friedreich’s Ataxia

Friedreich’s Ataxia (FRDA) is a rare autosomal recessive genetic, multi-faceted disease. It is characterized by progressive neurodegeneration and hypertrophic cardiomyopathy caused by severely reduced levels of frataxin, a mitochondrial chaperone protein involved in iron–sulfur cluster assembly [89]. Frataxin is indispensable for the incorporation of iron into iron–sulfur clusters. Iron–sulfur clusters are ancient protein cofactors—in eukaryotes, they are present in both mitochondria and cytosol—that have a critical role in essential biological pathways including cellular respiration, protein translation, and neurotransmission [90]. It has been observed that frataxin deficiency results in iron accumulation within mitochondria and increased sensitivity to oxidative stress. One of the consequences of the resulting redox stress in FRDA is the increase in the GS-ylation of actin, one of the most abundant cytoskeletal proteins. It has been observed that actin GS-ylation contributes to compromised microfilament organization in FDRA fibroblasts, resulting in impaired cytoskeletal functions [91]. The microtubule dynamics in an in vitro frataxin-silenced neuronal model of FRDA was also analyzed [92]. Microtubules play a key role in the physiology of neurons and any change in their dynamicity leads to an alteration of neuronal functions. It has been hypothesized that oxidative stress induces axonal retraction by interfering with microtubule dynamics. A decrease in polymerized microtubules in silenced motor neurons was associated with α-tubulin GS-ylation. In accordance with the reversibility of the oxidative injury, the authors suggested the possibility of a therapeutic approach to recover microtubule impairment by antioxidants [92].

#### 4.3.7. GS-Ylation in Microglia Cells

Microglia are resident immune cells that act as the first active defense in the central nervous system [93]. In the physiological state, microglia maintain a relatively quiescent monitoring state (M0 phenotype) and play an immune surveillance and defense role. Under pathological conditions, microglia—as a function of different stimuli—are activated to exert neuroprotective effects (M2 phenotype) or to release inflammatory factors and neurotoxic substances (M1 phenotype). Microglia overactivation towards the M1 phenotype is one of the driving forces of neurodegenerative disorders. Hypoxic conditions activate the M1 phenotype resulting in oxidative stress and the release of pro-inflammatory cytokines. The signal transducer and activator of transcription 1 (STAT1)—of the JAK/STAT signaling pathway—is activated by the M1 pathway and has a key physiological role in the regulation of the inflammatory response as well as in apoptosis. Oxidative stress induces both phosphorylation and the GS-ylation of STAT1 causing the hyper-activation of its signaling in microglia cells, pointing out the importance of this transcription factor in neuroinflammation in several neurological diseases [94,95]. The JAK/STAT signaling pathway constitutes a rapid signaling module from cell membrane to the nucleus and activates the expression of various critical mediators involved in cellular processes, including the modulation of immune and inflammatory responses. Dysregulation of the JAK/STAT pathway may result in various immune disorders and is associated with various cancers.

#### 4.3.8. Leber Hereditary Optic Neuropathy

Leber hereditary optic neuropathy (LHON) is a hereditary mitochondrial neurodegenerative disease of the optic nerve. LHON is one of the most common mitochondrial diseases and it involves a two-step process: an initial dysfunction of retinal ganglion cells, followed by a degeneration of their axons [96]. Mitochondrial DNA mutations cause a defective Complex I—the first enzyme of the respiratory chain—resulting in damaged electron transport chain activity. An overproduction of ROS due to Complex I deficiency in cells carrying LHON has been observed, and this oxidative stress has been related to enzymic dysfunction caused by the increased GS-ylation of different proteins [97]. Using a proteome-wide approach, it has been shown that, among the proteins with increased GS-ylation levels, only 27% were mitochondrial proteins, while the others had different extra-mitochondrial localizations suggesting that the disease is not associated exclusively to the mitochondrial environment but has a wider harmful impact throughout the cell [97].

The role of GS-ylation in mitochondrial homeostasis, and how the deregulation of this modification is associated with neurodegenerative disorders, has already been the subject of excellent reviews to which we refer the reader [16,98,99].

### 4.4. Kidney Diseases

In the kidney, GS-ylation has been shown to be involved in various physiological and pathological processes [100]. It is implicated in the regulation of renal oxidative stress, inflammation, and apoptosis. In kidney disease, impaired Nrf2 activity due to altered GS-ylation can lead to the exacerbation of oxidative damage and inflammation. The GS-ylation of specific proteins in the kidney can modulate their activity and function, influencing renal function and homeostasis. One example of the importance of GS-ylation in the kidney is its role in regulating the activity of the sodium–potassium ATPase (Na^+^/K^+^ ATPase), which is responsible for maintaining the balance of sodium and potassium ions in renal cells [100]. The GS-ylation of the Na^+^/K^+^ ATPase can affect its activity, thereby influencing renal salt and water reabsorption. GS-ylation has also been implicated in the pathogenesis of various kidney diseases, including diabetic nephropathy, renal fibrosis, and acute kidney injury. In these conditions, excessive oxidative stress and impaired redox balance can lead to the increased GS-ylation of proteins, contributing to renal damage and dysfunction [100]. It has been observed that GS-ylated hemoglobin was detected at higher concentrations in hemodialysis and continuous ambulatory peritoneal dialysis patients than normal subjects [101]. Thus, the measurement of GS-ylated hemoglobin may be useful to evaluate oxidative stress in uremic patients.

#### GS-Ylation of Cystathionine β-Synthase

Homocysteine (Hcy) is a non-proteinogenic sulfur amino acid whose metabolism is at the intersection of two metabolic pathways: remethylation to form methionine and trans-sulphuration to form cysteine, the limiting factor in the synthesis of GSH [102]. Accumulation of Hcy has been indicated as a risk factor for several disorders, such as cardiovascular and neurodegenerative diseases, cancer, chronic kidney disease, and other diseases [103]. Kidney is the major site of Hcy metabolism with serum Hcy levels in patients with chronic kidney disease being higher than those in patients with normal renal function [104]. The main cause of hyperhomocysteinemia is a redox imbalance leading to oxidative stress characterized by an accumulation of oxidized GSH followed by an impairment of cellular integrity and kidney homeostasis. Cystathionine β-synthase (CBS) is the first enzyme in the trans-sulphuration pathway in Hcy metabolism [102]. CBS is also involved in alternative reactions that result in the biogenesis of hydrogen sulfide, an important gaseous signaling molecule with cytoprotective effects [105]. It was demonstrated that the GS-ylation of CBS at residue Cys346 enhances its enzymatic activity when the cells were subjected to oxidative stress, increasing the synthesis of cysteine and then GSH and hydrogen sulfide [106]. These results suggest a model for an autocorrective cellular response to a decrease in GSH levels due to oxidative stress.

### 4.5. Lung Disease

In the lungs, oxidative stress can occur due to exposure to environmental pollutants like cigarette smoke, air pollutants, and respiratory infections. Oxidative stress can lead to inflammation, tissue damage, and the development of lung diseases such as chronic obstructive pulmonary disease, asthma, and lung cancer.

#### Idiopathic Pulmonary Fibrosis

Idiopathic pulmonary fibrosis (IPF) is the most common type of pulmonary fibrosis and the lung damage from IPF is irreversible and progressive. IPF is characterized by the loss of the bronchiolar and alveolar epithelial cells concurrent with accumulation of myofibroblasts. GS-ylation is implicated in the pathogenesis of IPF. It has been demonstrated that proapoptotic Fas receptor is a key mediator in increasing fibrosis [107]. The activation of Fas receptors—by the binding of Fas ligands expressed by myofibroblasts—triggers epithelial cell apoptosis. It has also been observed that the Fas ligand promotes the GS-ylation of the Fas receptor with the subsequent amplification of epithelial cell apoptosis. GSTP—an enzyme involved in GS-ylation—is the most abundant isoenzyme of the GST class in the human lung [108]. Inhibition of the enzymatic activity of GSTP attenuated fibrotic remodeling in two mouse models of pulmonary fibrosis, partially, through a mechanism involving Fas-SSG [107]. Thus, GSTP inhibition may be an effective target for the treatment of IPF and other lung fibroproliferative diseases.

### 4.6. Liver Disease

#### GS-Ylation of Notch1

Alcohol-related liver disease refers to liver damage caused by excessive and prolonged alcohol consumption. There are several stages of severity and a range of associated symptoms, resulting in liver failure up to liver cancer. Alcohol-induced oxidative stress is correlated to the metabolism of ethanol that is directly involved in the production of ROS and RNS [109]. Chronic alcohol use consumption significantly impacts on innate and adaptive immune responses involving several cells including macrophages that are essential in the progress of alcohol-induced liver inflammation. Scavenger receptor A (SRA) is a cell surface glycoprotein mostly expressed in macrophages which is known to participate in multiple macrophage metabolic processes, including adhesion, phagocytosis, and host defense [110]. Notch signaling is a highly conserved cell signaling pathway that is crucial for development and homeostasis in multicellular organisms, including mammals. It is involved in various biological processes, including stem cell maintenance, tissue regeneration, and immune system function. Aberrant Notch signaling has been implicated in numerous cancerous and noncancerous diseases. In relation to cancers, Notch signaling can both promote and inhibit tumor development in various types of cancers [111]. It was also reported that alcohol induces the Notch pathway activation. Recently, it has been observed that, under alcoholic exposure, SRA deficiency in a mouse model (SRA^-^/^-^) intensified hepatic injury due to the increased inflammatory response [112]. In SRA-deficient hepatic macrophages, increased inflammation was associated with enhanced Notch1 signaling pathway activation through the GS-ylation of Notch1. SRA mediated the negative regulation of Notch activation through interactions with thioredoxin, facilitating the binding of the enzyme to Notch1. At the molecular level, SRA directly bound Notch1 and suppressed its GS-ylation, thereby inhibiting the Notch pathway activation [112]. It has been suggested that SRA-mediated negative regulation of Notch activation might serve as a novel therapeutic approach for subjects with alcohol-induced liver injury.

Liver fibrosis derives from chronic damage to the liver in conjunction with the excessive accumulation of extracellular matrix (ECM) proteins—such as collagen—which causes damage to the liver tissue and hampers its ability to function properly. Hepatic fibrosis is a progressive condition that, if untreated, can develop into cirrhosis which could be followed by liver failure and hepatocellular carcinoma. The activation of hepatic stellate cells (HSCs), identified as the major source of ECM in the injured liver, is the dominant event in liver fibrosis. The activation of HSCs is due to several intracellular and extracellular events and signals, including oxidative stress and transforming growth factor β—Smad3 pathway activation. The activation of Smad3 supports the transcription of type I and type III collagen [113]. ROS produced by damaged liver cells cause paracrine activation signals to HSCs [113]. It was showed that pirfenidone, an anti-lung fibrosis drug, inhibited HSC activation and liver fibrosis in a Grx-dependent manner [114]. Instead, during pirfenidone treatment, there was a significant induction of the oxidoreductase enzyme. Smad3 has been identified as a Grx substrate, and the GS-ylation of Smad3 is required for its fibrogenic activity. The inhibition of HSC activation by Grx has been explained through the de-GS-ylation of Smad3, which prevents Smad3 phosphorylation, leading to the suppression of fibrogenic gene expression [114]. Thus, both Grx and the GS-ylation of proteins could be used as biomarkers. In addition, Grx induction by pirfenidone could be a promising strategy to prevent and treat liver fibrosis.

### 4.7. Cancer

GS-ylation can influence the initiation and progression of cancer, being involved in a wide range of processes [115]. Furthermore, its important role in anti-cancer drug resistance has been observed [115]. Cancer is a condition characterized by unchecked cell proliferation due to the excessive activation of cell division and the corresponding suppression of apoptosis. The hyperproliferation of tumor cells is accompanied by the production of high ROS levels capable of influencing cancer evolution [116]. Tumor cells tolerate these high ROS levels by increasing their antioxidant status to maximize ROS-driven proliferation, while at the same time avoiding senescence, or programmed cell death induced by the ROS [116]. The increase in the production of intracellular ROS is due to various mechanisms including the intensification of ATP production to support the rise in cellular components, alterations in the signaling pathways associated with cancer metastasis, and to the hyper activation of several oncogenes [115]. Some examples of how GS-ylation may contribute to such processes are given below.

#### 4.7.1. GS-Ylated NF-κB

Nuclear factor-kB (NF-κB) consists of a family of transcription factors—expressed in almost all cell types and tissues—that plays critical roles in inflammation, immunity, cell proliferation, differentiation, and survival [117]. NF-κB contributes to malignancy through impacting cell senescence, apoptosis, metabolism, stress responses, and tumorigenesis. In non-small cell lung cancer (NSCLC)—one of the two main types of lung cancer—NF-κB is highly expressed in comparison with normal tissue. Its activation, induced by external stimuli, regulates the expression of several downstream pathways, thereby promoting cancer cell proliferation and survival [118]. Furthermore, high levels of NF-κB are associated with adverse prognoses. GS-ylation is involved in modulating NF-κB activity and the consequent expression of downstream genes [118]. GS-ylation controls the activation of the transcription factor at multiple levels. It has also been observed that the GS-ylation of NF-κB correlated with enhanced lung inflammation in response to oxidants. Overall, the GS-ylation of NF-κB would be symptomatic of NSCLC and therefore, could be used as a prognostic biomarker in the future [118].

#### 4.7.2. GS-Ylated PKC

Protein kinase C (PKC) is a family of serine-threonine kinases involved in signaling pathways, thereby regulating several cellular processes, including growth, proliferation, differentiation, and apoptosis [119]. PKCs are ubiquitously expressed in tissues. Dysregulation of PKC signaling has been implicated in various diseases, including cancers [119]. Different expression and activity of PKCs has been associated with several types of cancers. PKC isoenzymes may display alterations in expression during cancer progression, but abnormal expression of other isozymes can occur too. As an example, PKCε overexpression is a prominent feature of human prostate cancer [120]. On the other hand, it has been observed that the GS-ylation of PKCε results in the complete inactivation of the enzyme [121]. PKCs have cysteine-rich regions that are highly prone to oxidation [5]. The complete inactivation of PKC by GS-ylation has been observed also for other isozymes [5,122]. In short, it has been deduced that the antagonistic role of GSH in cancer progression is mediated by the oxidative inactivation of PKC isozymes.

#### 4.7.3. GS-Ylated p53

The talent of p53 to maintain genomic stability is due to its ability to stop the proliferation of cells with damaged DNA, which reduces the risk of tumor development. It has been demonstrated that over 50% of human cancers is characterized by the occurrence of loss of function mutations in the *TP53* gene [123]. Cancers with mutations or deletions in the *TP53* gene are characterized by uncontrolled cell growth, aggravated metastasis, and increased drug- and radiation-resistance. In normal cells, p53 has a short half-life of about 20 min, while that of mutant p53 is prolonged up to 12 h [123]. It has been shown that human p53 is inhibited by the GS-ylation of three cysteines all present in the proximal DNA-binding domain during oxidative stress with Cys141 being the most reactive of them and representing the major site for GS-ylation [124]. Polyclonal antibodies against Cys141-GS-ylated p53 were developed to obtain an efficient biomarker that could be used in several diseases including cancer [125]. Antibodies exclusively recognized Cys141-GS-ylated p53 but not any other GS-ylated proteins. Their efficacy was positively tested in different types of cancers.

**Table 1:** GS-ylation effects associated with several pathologies.

Pathological Conditions	Target Protein	Function	Functional Impact/ Effect of GS-Ylation	Ref.
**Cardiovascular disease**				
Diastolic dysfunction	cMyBP-C	Cardiac contraction and relaxation	Heart failure	[42]
Vascular barrier dysfunction	Rac1	Correct function of the cell barrier	Cell hyperpermeability	[48]
**Cerebrovascular disease**				
Different and multifactorial disorders of the blood vessels	eNOS	Prominent enzymatic source of NO in the vascular wall	Functional uncoupling, reduction of NO synthesis, increased $O_{2}^{-}$	[49]
**Vascular disease**				
Maintaining the endothelial barrier function	Src tyrosine kinase	Phosphorylation of VE-cadherin	Inhibition of phosphorylation	[53]
Marfan syndrome	SirT1	Prevention of aortic dissections	Contributing to thoracic aortic aneurysm in Marfan syndrome	[55]
**Neurodegenerative disease**				
**Alzheimer’s**				
Slowdown of glycolysis	GAPDH	Glycolytic enzyme	Inhibition of enzymatic activity	[64]
Inhibit functionality of p53	p53	Control the expression of a wide array of genes	Prevents the formation of the tetramer form of p53	[70]
**Parkinson’s**				
Positive feedback regulatory mechanism	KEAP1	Regulator of Nrf2 activity	Nrf2 activation and subsequently expression of its augments	[75]
**Huntington’s**				
Striatal neuron loss	TRPC5	Calcium channels	Enhancement of calcium ions in cytosol	[81]
**Amyotrophic lateral sclerosis**				
Excessive release of ER calcium into cytoplasm	STIM1	Maintain cellular Ca^2+^ balance	Dysregulation of Ca^2+^ entry	[82]
**Friedreich’s ataxia**				
Impairment of cytoskeletal functions	Actin	Cytoskeletal proteins	Compromission of microfilament organization in FDRA fibroblasts	[91]
Neuroinflammation	STAT1	Regulation of inflammatory response	Hyper-activation of its signaling in microglia cells	[95]
**Other organ diseases**				
**Kidney**				
Influencing renal salt and water reabsorption	Na^+^-K^+^ ATPase	Maintains the balance of sodium and potassium ions in cells	Affect pump activity	[100]
**Lung**				
Implicated in the pathogenesis of IPF	Fas receptor	Trigger epithelial cell apoptosis	Amplification of epithelial cells apoptosis	[108]
**Liver**				
Increased inflammation	Notch1	Involved in various biological processes	Enhanced of Notch1 signaling pathway	[112]
**Cancer**				
NF-κB highly expressed in NSCLC	NF-κB	Involved in various biological processes	Enhanced lung inflammation	[118]
Dysregulation of PKC signaling	PKC	Involved in signaling pathways	Complete inactivation of the enzyme	[121]
Inhibit functionality of p53	p53	Control the expression of a wide array of genes	Complete inactivation of transcription factor	[124]

#### 4.7.4. GS-Ylated BiP

Multiple myeloma is a blood cancer characterized by the accumulation of monoclonal plasma cells in the bone marrow, resulting in excess production of monoclonal immunoglobulin [126]. The ubiquitin–proteasome system (UPS) catalyzes the degradation of ubiquitinated intracellular proteins playing a key role on regulation of cell proliferation and survival [127]. The inhibition of UPS results in the perturbation of intracellular protein homeostasis—fundamental for cell function and viability—by the accumulation of poly-ubiquitinated proteins, which subsequently induces cellular stress and apoptosis. Cancerous cells appear to display a higher dependence on the UPS than normal cells and therefore, UPS inhibition may have a therapeutic value. Indeed, bortezomib is a first-in-class proteasome inhibitor used to treat multiple myeloma. However, prolonged treatment with bortezomib resulted in drug resistance. It has been observed that resistant cells maintain a high level of oxidative stress and express high levels of GSTP. Its localization in the lumen of the endoplasmic reticulum enables the GS-ylation of resident proteins such as the binding immunoglobulin protein (BiP), a critical player of the unfolded protein response. Recently, it has been demonstrated that the mechanism of acquired bortezomib resistance is due to the high levels of the GSTP enzyme produced under oxidized conditions [128]. As an example, the GS-ylation of BiP by GSTP increases the efficiency of BiP in protein folding, thereby increasing the ability of cancer cells to tolerate the drug [128].

## 5. GS-Ylated Proteins as Biomarkers

During constitutive metabolism, the levels of GS-ylated cellular proteins are at least an order of magnitude lower than those in cells under oxidative stress. Thus, the detection of GS-ylated proteins in biological samples can provide insights into the redox status and cellular damage, making it a potential biomarker for disease diagnosis and monitoring treatment efficacy. Several techniques may be used to measure GS-ylation both qualitatively and quantitatively. As example, an analytical approach for the determination of S-glutathionylated proteins based on the specific reduction of mixed disulfides by Grx, their reaction with N-ethylmaleimide–biotin, followed by affinity purification on avidin-agarose of tagged proteins and identification by proteomic analysis has been developed [129]. This method was subsequently improved by analyzing and identifying eluted proteins by liquid chromatography and quadrupole time-of-flight tandem mass spectrometry (LC-MS/MS) [130]. Recently, a protocol using the fluorescent probe monobromobimane to label GSH after its release from mixed disulfides by reduction with dithiothreitol and quantified by HPLC has been reported [131]. This method can be applied to many different biological samples, comprising blood components, red blood cell plasma membranes, cultured cells, and solid organs from animal models [131]. To measure GS-ylated proteins, a quantitative multimeric immunoprecipitation method was also used (see paragraph on the GS-ylation of cMyBP-C) [42]. GS-ylated hemoglobin increases in several human diseases and it could be used as a clinical biomarker of oxidative stress (see paragraph on Kidney Diseases and [132]). In patients with type 2 diabetes mellitus, the concentration of GS-ylated hemoglobin—analyzed by liquid chromatography/electrospray ionization mass spectrometry (LC/ESI-MS)—was significantly elevated in their plasma compared with healthy subjects [133]. GS-ylated hemoglobin, used as a biomarker of oxidative stress by MALDI-TOF-MS, was also measured in heavy smokers and in occupational obese subjects [134]. The biotin switch assay has also been used. Furthermore, a new procedure to analyze GS-ylation using the MS-based biotin switch assay has been described together with several other examples [135].

## 6. Therapeutical Applications

In a therapeutical approach, targeting GS-ylation can have several potential applications, such as for anti-cancer therapy, for the treatment of IPF, in liver injury, and in neurodegenerative diseases [63,92,107,112,124,136]. In neurodegenerative and cardiovascular diseases, modulating the GS-ylation process could potentially help prevent or slow down disease progression [63,92,136]. In anti-cancer therapy, some studies suggest that the GS-ylation of specific proteins can promote tumor growth and survival. Targeting these GS-ylation sites may provide new therapeutic strategies for cancer treatment [124]. GS-ylation plays a crucial role in the detoxification of drugs and xenobiotics in the body. Manipulating (controlling) GS-ylation pathways may enhance drug efficacy, reduce toxicity, or increase the elimination of harmful substances [21,35,116,137,138].

Disulfiram, a drug clinically used in the treatment of chronic alcoholism, has been found to have anti-cancer activity [139,140]. Disulfiram is a lipophilic dithiocarbamate that crosses the cellular membrane and complexes with metal ions. These complexes disrupt essential signaling pathways through several mechanisms including the formation of ROS, ultimately inducing apoptosis. It was hypothesized that a disulfiram copper gluconate combination would inactivate key oncoproteins important for malignant cell growth through the GS-ylation of serum proteins [141]. The study was carried out on patients with advanced solid tumors to the liver. The liver is a common site of metastasis for a variety of solid tumors, most commonly adenocarcinomas of the colon or pancreas. The increased GS-ylation of serum proteins was observed with treatment, suggesting that this drug combination could exert a suppressive effect on cellular growth and protein function. Moreover, it has been suggested that protein GS-ylation could be used as a pharmacodynamic biomarker [141].

Overall, therapeutically targeting GS-ylation represents a promising approach for the treatment of various diseases and conditions. However, more research into the specific mechanisms and targets of GS-ylation is needed to fully elucidate its therapeutic potential.

## 7. Conclusions and Future Perspectives

The survey of the recent literature on protein GS-ylation that we have presented here clearly shows that, in the context of human diseases, this post-translational modification plays a significant role, especially because of its connection with oxidative stress and inflammation. Indeed, GS-ylation impacts on protein function at many levels, from enzymatic activity to protection from oxidative modifications, manipulation of proteins’ interaction properties and protein stability. As such, it is implicated in the onset of several serious diseases, ranging from cardiovascular to neurodegenerative, cancer, and many others. Many advances in this research field have already been achieved, but it is not difficult to foresee that further future research will enable a more precise mapping of GS-ylation sites in different conditions and the detection of disease specific modifications that will provide insights into disease mechanisms and progression. This, in turn, will have many implications for the implementation of new biomarkers, based on the detection and quantification of specific glutathionylated proteins, useful both for the early diagnosis and monitoring of disease progression. Ultimately, we have provided a few examples of how the manipulation of GS-ylation, for instance by the inhibition of GSTP, the main enzyme catalyzing this reaction, may be useful for therapeutic purposes in selected cases. It is not too ambitious to hypothesize that, as our understanding of the molecular mechanisms underlying GS-ylation improves, this knowledge could lead to novel, effective treatments for a range of human diseases.
